# A Review of 10 Years of Vasectomy Programming and Research in Low-Resource Settings

**DOI:** 10.9745/GHSP-D-16-00235

**Published:** 2016-12-23

**Authors:** Dominick Shattuck, Brian Perry, Catherine Packer, Dawn Chin Quee

**Affiliations:** a Georgetown University's Institute for Reproductive Health, Washington, DC, USA.; b Duke Clinical Research Institute, Durham, NC, USA.; c FHI 360, Durham, NC, USA.

## Abstract

Vasectomy is a highly effective and safe contraceptive method for couples who want to stop childbearing, but only 2.4% of men around the world use this method. We conducted an extensive review of the vasectomy research literature and programmatic reports, published between April 2005 and April 2015, to synthesize barriers and facilitators to vasectomy adoption. Of the more than 230 documents initially retrieved in our search, we ultimately included 75 documents in our review and synthesized the findings according to the Supply–Enabling Environment–Demand (SEED) Programming Model. Regarding promoting demand for vasectomy services, we found there was a general lack of awareness about the method among both men and women, which often fueled erroneous assumptions about how vasectomy affects men. Several types of programmatic activities directly addressed knowledge gaps and negative misperceptions, including community-based and mass media communications, employer-based promotion, and group counseling. For supply of services, the lack of or inaccurate knowledge about vasectomy was also prevalent among providers, particularly among community-based health workers. Programmatic activities to improve service delivery included the use of evidence-based vasectomy techniques such as no-scalpel vasectomy, whole-site trainings, task shifting, cascade training, and mobile outreach. Finally, programmatic approaches to building a more enabling environment included engagement of governments and other community and religious leaders as well as campaigns with gender transformative messaging that countered common myths and encouraged men's positive engagement in family planning and reproductive health. In summary, a successful vasectomy program is comprised of the mutually reinforcing components of continual demand for services and access to and supply of well-trained providers. In addition, there is an underlying need for enabling policies within the cultural and gender environments that extend beyond vasectomy and include men not just as default partners of female family planning clients but as equal beneficiaries of family planning and reproductive health programs in their own right. Accelerating progress toward meaningful integration of vasectomy into a comprehensive contraceptive method mix is only possible when political and financial will are aligned and support the logistical and promotional activities of a male reproductive health agenda.

## Abstract (precis)

Reviewed areas included misconceptions and lack of knowledge among men, women, and providers; approaches to demand generation including community-based and mass media communications; service delivery innovations consisting of the no-scalpel vasectomy technique, whole-site training, cascade training, task shifting, and mobile outreach; and engagement of religious and community leaders to create an enabling environment.

## BACKGROUND

Over the last several decades, national family planning initiatives have led to significant gains in many developing countries as exemplified through improvements in key Family Planning 2020 (FP2020) indicators.^1^ The initiatives continue to expand the quality of and access to family planning services, predominantly for women. More recently, research and programs that engage men in family planning and that combat inequitable gender norms have also increased in effectiveness and scope. A search of the abstracts accepted to the 2015 International Conference on Family Planning with the term “male involvement” resulted in 49 presentations across a variety of aspects that included improving couple communication, improving service delivery for men, and looking for new ways to increase male involvement in family planning.^2^ With this growing support and refinement of gender and male involvement programming, now is an opportune time to incorporate voluntary vasectomy services into national family planning strategies.

Research suggests that demand for permanent methods may increase over time as the age when women desire to limit family size (that is, to stop childbearing) continues to decrease.^3^ Analysis of Demographic and Health Survey data from 18 countries between 2004 and 2010 found that the demand to limit births began to exceed the demand for spacing births, on average, at 33 years old. In some countries, however, the desire to limit births predominated at an age as low as 23 years.^3^

When a couple desires to limit their family size, the most effective methods with the least side effects should be available. Vasectomy is one of these methods but is used little around the world. On the other hand, female sterilization (tubal ligation) is the most commonly used form of contraception worldwide: 19% of women in union are sterilized versus 2.4% of men globally.^4^ This is despite the fact that vasectomy has no side effects and, compared with female sterilization, is less risky of a procedure, provides a quicker recovery period, and costs the health system less per client. The correlation between the use of female sterilization and vasectomy is complex, as less developed countries contribute to the highest use of female sterilization but have the lowest prevalence of vasectomy.

Vasectomy is one of the most effective contraceptive methods with no side effects but is little used around the world.

Many other couples depend on short-acting methods (e.g., condoms, pills, injectables) to limit their births, which, when compared with long-acting or permanent methods (LAPMs), have greater costs for both governments and clients (time and money), are less effective due to potential product failure, and have higher rates of discontinuation and/or incorrect use.^5^

Vasectomy, however, could be a viable option for many couples. Providers across the globe have been trained to perform no-scalpel vasectomies (NSV). This method requires only a small puncture in a man's scrotum to access the vas deferens, with the client under local anesthesia. NSV has been found to be the preferred technique by physicians for isolating and accessing the vas deferens.^6^^–^^9^ Cauterization of the lumen of the vas deferens combined with fascial interposition results in the lowest risk of occlusive failure (well below 1%, according to post-vasectomy semen analysis).^7^^,^^8^ This technique is already widely used in North America.^10^ Recently, it was integrated within all district hospitals across Rwanda,^11^ suggesting that providers in low-resource settings can be trained in this method and that training in supplemental NSV with advanced occlusion (e.g., fascial interposition and thermal cautery) can maximize the effectiveness of ongoing vasectomy programs in low-resource settings.^12^

In this article, we review recent literature related to voluntary vasectomy programs in low-resource settings to synthesize common barriers and facilitators to vasectomy uptake and identify recommendations to strengthen future vasectomy promotion efforts.

## METHODS

In April 2015, we conducted a search of both the peer-reviewed and gray literature using 8 search engines: POPLINE, PubMed, Global Health, Cumulative Index to Nursing and Allied Health Literature (CINAHL), Africa-Wide Information, Academic Search Premier, Google Scholar, and the United States Agency for International Development's (USAID's) Development Experience Clearinghouse. Keywords used in the search were as follows: vasectomy OR “male sterilization” AND accept* OR “communication strategy” OR “contraceptive methods chosen” OR counsel* OR “delivery of health care” OR demand OR evaluat* OR “health services” OR implement* OR intervention* OR introduce* OR messaging OR program* OR promot* OR “scale up” OR “scaling up” OR “social marketing” OR success OR uptake. To limit our search to the most current and relevant literature, our inclusion criteria included documents published in English within the last 10 years (April 2005 to April 2015). We excluded documents describing vasectomy programs from Australia, Canada, the United Kingdom, or the United States. It is possible that some important resources published prior to April 2005 may not be reflected in this current review.

Our search retrieved more than 230 documents, of which approximately two-thirds were excluded because they were duplicates or did not meet our criteria. Two analysts categorized the remaining 75 documents according to their subject matter. We created matrices in Microsoft Excel to summarize and synthesize the content of the documents in each category, to highlight important barriers to and facilitators of vasectomy uptake, and to highlight key recommendations for future vasectomy programs. Finally, we applied the Supply–Enabling Environment–Demand (SEED) Programming Model to present the key findings from the 75 documents we reviewed. (See the supplementary material for a table of all 75 documents organized by region of the vasectomy program or research.) The SEED model has been established as a useful global framework for sexual and reproductive health programming.^13^ It is based on the principle that programs will be more successful and sustainable if they address the multifaceted determinants of health and if they include interventions that simultaneously (1) address the availability and quality of services and other **supply**-related issues, (2) strengthen the health system and foster an **enabling environment** for healthy sexual and re-productive health behavior, and (3) improve knowledge of sexual and reproductive health and promote **demand** for services. The SEED domains are—by design—overlapping and interrelated, as programmatic activities designed to address deficiencies in one domain can often improve conditions in other domains as well.

We reviewed 75 documents from the peer-reviewed and gray literature on vasectomy programs.

Information gathered from this review has been published in a final report for USAID and has been used to inform the development of 8 country-specific advocacy briefs (https://www.fhi360.org/resource/promoting-evidence-based-vasectomy-programming).

## FINDINGS

### Demand for Vasectomy Services

To be motivated to use vasectomy services, an individual or couple first needs accurate knowledge of and positive attitudes toward vasectomy. Potential vasectomy clients must also know where services are available, understand details about the procedure (e.g., side effects, recovery time, and time required for back-up contraception), and believe that services are confidential. Below, we outline barriers and facilitators related to demand for vasectomy services.

#### Barriers to Promoting Demand for Vasectomy

**Lack of knowledge.** Much of the literature we reviewed indicated there was a general lack of awareness about vasectomy and lack of basic knowledge about the procedure among prospective clients (both men and women), posing a major initial demand-promotion barrier. In 5 studies from Ethiopia, Nigeria, and Turkey,^14^^–^^18^ awareness of vasectomy as a family planning method ranged from 15.6% of Ethiopian women^14^ to 39.6% of unmarried Turkish men.^16^ However, awareness of vasectomy was high in India (97.4%)^2^ and Nepal (77%).^19^ Still, basic knowledge of how the procedure is conducted, requirements related to follow-up, or side effects from the procedure was still lacking across sites and studies.^19^^–^^23^ Disparities between men's and women's knowledge of vasectomy were rarely discussed in the literature. Among the few exceptions were two qualitative studies, from Malawi^24^ and Nigeria,^25^ that found that men were less knowledgeable than women about family planning methods in general and about LAPMs specifically.

Men and women generally lack knowledge about vasectomy, posing a major demand-promotion barrier.

**Negative attitudes.** Inaccurate knowledge often fueled erroneous assumptions about how vasectomy affects men physiologically and psychologically.^17^^,^^18^^,^^20^^–^^33^ In some studies, participants perceived that vasectomy hurt a man's pride^34^ or caused a man to lose his “masculinity.”^35^ Men worried that others would view them negatively if knowledge of their vasectomy was public.^22^^,^^36^ In Ghana^23^ and India,^22^ participants felt that if a man got a vasectomy he would be viewed as “under the control of” or a “slave to” his wife. Another Indian study found that women preferred female sterilization over vasectomy because they felt it was better for a woman (than a man) to be “debilitated” since the economic contributions of men were more highly valued than those of women.^32^ A number of studies mentioned negative attitudes about the method because people thought vasectomy would lead to male infidelity^23^^,^^29^^,^^30^ or an inability to perform sexually,^36^ and some women feared men would retaliate or reject the possibility of method failure, resulting in negative consequences for women.^22^

Inaccurate knowledge often fuels erroneous assumptions about how vasectomy affects men physiologically and psychologically.

**Low intention to use.** In most of the documents reviewed, prospective clients' willingness to use vasectomy was very low, due in large part to limited knowledge and negative attitudes. A Nigerian study found that only a small percentage of men reported even considering a vasectomy.^37^ Similarly, few Nigerian or Indian women viewed vasectomy as acceptable,^17^^,^^32^ or something their husband would choose.^32^ This is partially exemplified in an Indian study where 68% of men found vasectomy acceptable, but only 34% suggested they would adopt it.^20^

We should note that acceptability and use of contraception is not solely dependent upon client (or provider) knowledge and attitudes toward the method. Behavioral theories abound describing the multitude of factors that contribute to client acceptance (e.g., opportunity and financial costs, social norms, perceived need, etc.), but accurate knowledge and positive attitudes are fundamental to ensuring informed and voluntary use of any method or health care procedure.

#### Facilitators to Promoting Demand for Vasectomy

Although documentation of knowledge and attitudinal barriers abounded in the literature, references to important facilitating factors were also present, including perceived benefits of the procedure among men and women as well as demographic information about the expected va-sectomy client base. Programmatic activities that directly addressed knowledge gaps and rampant negative misperceptions toward vasectomy included community-based and mass media communications, an employer-based promotion intervention, and a group counseling approach.

**Perceived benefits.** Positive attitudes toward and perceived benefits of vasectomy—although mentioned in fewer than half of the articles reviewed—are important building blocks for increasing demand for services. Frequently cited benefits were related to the high contraceptive effectiveness of the method, clients' quick recovery time, and the comparative safety and lower costs associated with the vasectomy procedure versus tubal ligation.^18^^,^^20^^–^^22^^,^^24^^,^^25^^,^^29^^,^^30^^,^^37^ Men and women in Cambodia and Malawi reported the benefits of sharing family planning responsibilities.^24^^,^^36^ Tanzanian women suggested that vasectomy would eliminate the possibility of having a child out of wedlock.^29^^,^^30^ In addition, Brazilian,^38^ Indian,^22^ Rwandan,^39^^–^^41^ and Tanzanian^29^^,^^30^ men described how vasectomy was beneficial to preserving the health of women (e.g., by avoiding frequent pregnancies and negative impacts of other forms of contraception) and that it was considered a minor procedure compared with female sterilization. Hearing positive testimonials was one of the main drivers of positive attitudes toward vasectomy in India—women felt encouraged and men were more open to the procedure.^22^

Frequently cited benefits of vasectomy included high contraceptive effectiveness, quick recovery time, and comparative safety and lower costs compared with tubal ligation.

Overall, in articles related to vasectomy client perspectives, couples using vasectomy were satisfied with the fast recovery time and the maintenance of sexual function.^39^^,^^42^^–^^44^ Motivations leading to vasectomy uptake included the desire to limit births, limited financial resources (not being able to afford more children), concern for women's health (desire to avoid pregnancies, births, and contraceptive side effects), and dissatisfaction with other contraceptive methods.^29^^,^^30^^,^^33^^,^^39^^–^^42^^,^^45^^,^^46^ Persuasive sources of vasectomy information for men included health workers, peers, and satisfied clients.^31^^,^^32^^,^^39^^,^^43^^,^^45^^,^^47^^,^^48^ Men in Ghana,^49^ Rwanda,^41^ and Turkey^50^ typically reported having heard about vasectomy through the media or from health care workers, which helped them learn how to access services.

**Expected vasectomy clientele.** Vasectomy—and sterilization in general—is not an appropriate family planning option for everyone. Therefore, it may be valuable for vasectomy programs to understand who their expected client base is. Based on our review, couples using vasectomy were generally older (over 30 years old), were married or in union, had multiple children (often 4 or more) and more children than couples using reversible methods, and had a history of prior contraceptive use.^29^^,^^30^^,^^33^^,^^41^^,^^43^^,^^45^^,^^47^^,^^49^^,^^51^ However, socioeconomic levels, education levels, and numbers of children of vasectomy clients varied within and between regions.^45^^–^^47^^,^^50^^–^^52^ Previous contraceptive use among wives of vasectomy clients varied from a low of 37% in Pakistan^46^ to 59.2% in Turkey^50^ and 87% in Rwanda.^41^ It is important to note that the range of potential vasectomy clients is likely more diverse than current users and that there may be a growing demand for limiting births (and resulting unmet need) among other demographics. Van Lith et al.,^4^ for example, describe a landscape in which younger couples in sub-Saharan Africa are increasingly interested in limiting births.

Couples using vasectomy are generally older (over 30), are married or in union, have multiple children, and have a history of prior contraceptive use.

**Community-based and mass media communications.** Community-based and mass media communications can increase awareness and drive demand for vasectomy. The Capacity Project's pilot program in Rwanda developed robust communication materials to increase general knowledge and positive attitudes toward vasectomy. Communications strategies included outreach by community health workers (CHWs),^47^ formation of 12 va-sectomy support cooperatives for male clients, video testimonials of clients that were used in education and communication campaigns,^40^^,^^41^^,^^47^ and dissemination of strategic messaging through various media outlets, including radio, which informed potential clients of upcoming service days.^53^

The ACQUIRE Project led a vasectomy communication campaign called “Get a Permanent Smile” in several low-resource settings.^54^ The campaign countered pervasive myths and rumors about vasectomy using various media outlets such as posters and television broadcasts staggered to coincide with seasonal periods of high media attention (in Bangladesh) and television and radio ads complemented by an information “hotline” and community outreach (in Ghana).^55^ Spikes in demand for vasectomy were tied to the communication activities,^55^ which highlights the important link between mass media promotion and uptake of vasectomy services.

The “Get a Permanent Smile” campaign conducted in many low-resource settings resulted in spikes in demand for vasectomy.

**Employer-based promotion.** The RESPOND Project engaged men and promoted male involvement in reproductive health, including vasectomy, in 10 Indian workplaces. The companies involved in this 18-month employer-based health promotional campaign ranged from waste management to manufacturing to beverage bottling. Through the program, employees were allowed to attend health-related activities during normal working hours. Educational materials focused on LAPMs, and strategies included training industry-related health coordinators on LAPMs and interpersonal communication, positioning health desks in well-trafficked areas of the company, establishing health (including family planning) referral systems, and establishing a hotline for family planning referrals.^56^ Employees who participated in the campaign reported a stronger intent to use family planning and were more likely to have discussed family planning with their partners than employees who did not participate.^57^ Additionally, many existing family planning users switched from short-acting or traditional methods to LAPMs after participating in the intervention.

**Group counseling.** In the Philippines, a group counseling intervention promoted open discussion with couples about NSV, which resulted in increased knowledge and acceptability of vasectomy among potential users.^58^ The authors suggested that as participants interacted, argued, and agreed or disagreed about certain issues, they encouraged each other to try particular contraceptive methods. They noted that the advantage of having couples together in the session was that after being exposed to the same information about contraceptive methods, members of the couple were then able to discuss their own plans and make a decision together.^59^

### Supply of Vasectomy Services

Provision of high-quality vasectomy services must include adequate infrastructure, supplies, and equipment as well as well-trained, skilled, motivated, and supported staff. It is also important to have administrative, financial, and management systems in place that are accountable to the communities they serve.

#### Barriers to Vasectomy Service Delivery

**Lack of provider knowledge.** Lack of provider knowledge of vasectomy or inaccurate knowledge was a major service delivery barrier identified in the literature. In one publication, Nigerian physicians were reported to have good general knowledge of vasectomy as a permanent method, but some thought that it would impair a man's ability to ejaculate or would increase his risk for prostate cancer.^60^ Another study in Nigeria found that 90% of male health workers interviewed were aware of vasectomy, but they had varying degrees of knowledge as to whether local, general, or no anesthesia was used during the procedure.^26^ A qualitative study from Cambodia found that, in general, village-level providers had little or incorrect knowledge about LAPMs, including vasectomy.^36^ Two surveys conducted in India explored vasectomy knowledge of CHWs and found that there was a great deal of knowledge around a person's eligibility for vasectomy as well as how long the procedure typically takes, but little knowledge of the details of the procedure (i.e., whether NSV requires stitches, the amount of time a man would need to take away from work, and post-vasectomy contraceptive requirements). In addition, some CHWs erroneously believed that after vasectomy a man would lose physical strength, become weak or get sick often, would not be able to have an erection or ejaculate, and would have reduced libido^61^^,^^62^—many of the same misconceptions held by men and women in general. It is evident from these studies that more needs to be done to improve provider knowledge about vasectomy, particularly among community-based health workers on the front lines of the health system. Community-level staff often provide people with their first exposure to new services that are available in health centers; their clear understanding and buy-in of methods such as sterilization are essential to shaping the public knowledge and perceptions of vasectomy.

Lack of knowledge about vasectomy is also prevalent among providers.

**Negative attitudes among providers.** Two studies that we reviewed explored how family planning providers' negative attitudes toward vasectomy influenced their willingness to provide the method.^42^^,^^60^ Both studies described how some providers acknowledged counseling biases toward female sterilization and avoided counseling on vasectomy. Provider attitudes and individual perceptions of appropriate family planning methods for their culture (Nigeria)^60^ were juxtaposed against their fear of complications and limited financial gains from providing vasectomies (China).^42^

Acceptability of vasectomy among providers was split between professional acceptability (i.e., willingness to refer clients for vasectomy) and personal willingness to use the method themselves. For example, in 2 Nigerian studies, a minority of providers (19.2%) accepted vasectomy as a contraceptive method and less than half of those would consider using the method themselves (41.3%)^26^; none of the doctors (or their partners) in either study actually had had a vasectomy.

#### Facilitators of Vasectomy Service Delivery

Programmatic activities geared toward creating or improving vasectomy service delivery included the use of evidence-based vasectomy techniques, whole-site trainings, task shifting, cascade training, mobile outreach, and tools to assist in program planning.

**Evidence-based vasectomy techniques.** Each of the programs in our review trained providers in NSV, highlighting the practicality of using this method to access the vas deferens in low-resource settings. Various methods were used by the different programs for occluding the vas once exposed, but Labrecque et al.'s^63^ review and evaluation of Asian vasectomy programs noted that most vasectomies were performed with NSV and simple ligation and excision technique for vas occlusion. This may be true in many low-resource countries due to the paucity of vasectomy services; however, to date no thorough review has been conducted.

Most vasectomy programs in Asia use the no-scalpel vasectomy technique with simple ligation and excision for vas occlusion.

From 2003 to 2004, the ACQUIRE Project visited vasectomy centers in Bangladesh, Cambodia, India, Nepal, and Thailand to observe vasectomy techniques and to demonstrate the novel occlusion techniques using handheld, battery-powered cautery devices and fascial interposition.^9^ ACQUIRE also conducted interviews with key informants in each country to gauge interest in the use of thermal cautery and/or fascial interposition techniques. The fascial interposition technique was largely known and even taught in the Asian countries visited but was seldom performed in Bangladesh, India, or Nepal. Barriers cited for not adopting fascial interposition included insufficient surgical skills, the additional time needed to perform the technique, and that it was not mandatory by country standards. Providers in these countries showed interest in the use of thermal cautery for vas occlusion.^9^

**Whole-site training.** Beginning in 2005, FRONTIERS and local partners in Guatemala developed a systemic vasectomy introduction model for Ministry of Health hospitals and maternity clinics.^64^ The model involved training the entire health team—surgeons, nurses, receptionists, and others who might provide referrals—on the benefits, procedures, and side effects of vasectomy. This whole-site approach increased general knowledge about vasectomy for the site teams. However, in a post-training survey, knowledge gaps remained around post-procedure counseling guidelines and characteristics of potential vasectomy clients.^65^^,^^66^ After the end of this project, the ministry used the whole-site model to introduce services in 10 additional hospitals and maternities.^65^^–^^67^

Likewise, the ACQUIRE Project in Ghana offered whole-site trainings to establish “male-friendly” services, in which all health staff were trained in NSV counseling and services.^55^^,^^62^ The whole-site training resulted in staff being more receptive to offering men's health services, a better understanding of male anatomy, fewer misconceptions about vasectomy, and more comfort in talking to men about vasectomy.^55^ Other related research from Jharkhand, India, also found that training CHWs in NSV and male anatomy increased knowledge about the procedure and reduced misconceptions, which improved counseling for potential clients.^61^

**Task shifting.** Vasectomy is considered a quick and routine procedure in most instances, which can be a benefit to physicians in low-resource settings who are extremely busy. For this reason, the discussion of task shifting vasectomy responsibilities to lower-level providers is common. In our search, we found some examples of this discussion and changes in policies. For example, Trollip et al. (2009) studied the safety and efficacy of vasectomy provision by junior-level doctors in South Africa.^68^ Procedure times and complication rates for 479 vasectomies were analyzed to assess the capacity of the physicians to perform the procedure, although they were not compared with those of more senior staff. Average operating times decreased significantly over time, but complication rates did not increase. This study suggests that with training and experience even junior-level medical staff may be able to efficiently provide vasectomy services without compromising the safety and efficacy of the procedure.

With training and experience, junior-level medical staff may be able to efficiently provide vasectomy services without compromising safety and efficacy.

The low rate of complications in general for vasectomy clients suggests that more investigation is necessary to determine the appropriateness of task shifting this procedure. Alternatively, an indirect approach to increasing services is being implemented in Malawi, where long-acting methods, comprising intrauterine devices (IUDs) and implants, are provided by outreach staff.^69^ This task shifting allows CHWs to provide a wider array of services that, in turn, may afford more technically skilled providers greater availability to offer more permanent methods to clients who have reached their desired family size.^24^^,^^25^^,^^28^

**Cascade training.** To systematically and cost-effectively build the capacity of clinics and service providers, many of the vasectomy programs we reviewed relied on a cascade approach to training.^53^^,^^54^^,^^70^ With the cascade approach, a small group of motivated providers and health staff are identified and trained to provide vasectomy service training. Once trained, this cadre is then trained as trainers. Over time, opportunities are provided for them to diffuse the knowledge and training to other providers and staff during the life of the program and after it ends (Figure).

**FIGURE.**
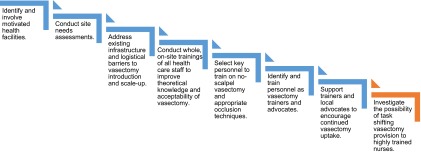
Cascade Training Model for Building Capacity in Vasectomy

Cascade training was implemented by the Capacity Project and PROGRESS in Rwanda. In both instances, the projects identified or developed curricula based on established procedures^11^^,^^71^^,^^72^ and created a skills checklist. This approach facilitated outreach visits by vasectomy teams from district-level hospitals to remote health centers to provide vasectomies and train other providers. Training under the PROGRESS Project took place over 5 days and included thermal cautery and fascial interposition. At the end of the training, the physicians successfully mastered the new occlusion technique.^11^ By 2012, the cascade training approach under the PROGRESS Project resulted in more than 64 physicians and 103 nurses trained in 42 hospitals, and 2,523 vasectomies were performed.

**Mobile outreach.** Mobile outreach services are often provided at static structures, in portable mobile health tents, or in vans.^73^ Our review identified several programs that used mobile outreach teams to expand the reach of vasectomy service provision. A key contribution to the success of the NSV program in Rwanda was the extension of service from hospitals to health centers. For example, 56% of vasectomies performed in a sample from one district were conducted at a rural health center as opposed to a district-level hospital.^40^

Padmadas et al. (2014) found that vasectomies were significantly more likely to be offered in a mobile clinic rather than a government hospital, particularly in remote locations.^73^ The Government of Nepal has mobilized outreach services for voluntary surgical contraception to rural areas of Nepal. Trained surgical teams travel to remote areas from a central location with necessary supplies. In locations where health facilities are not available, temporary settings such as schools and community centers are used.^69^ Wickstrom and colleagues from the RESPOND Project noted that community mobilization engages communities in discussing family planning; informs clients about all methods, including LAPMs; and ensures enough of a caseload of LAPM clients to make the outreach visit worthwhile.^69^

**Tools to assist in program planning.** We identified a handful of tools created to assist vasectomy program planners when integrating vasectomy services.^71^^–^^76^ (This is not a comprehensive list of all available tools and training curricula related to vasectomy due to the search criteria used in our study.) The ACQUIRE Project developed 2 training curricula that were designed to instruct physicians and vasectomy assistants to provide safe and effective NSV services.^71^^,^^72^ One document includes curricula on counseling clients; verifying informed decision making and consent; preventing infection and managing complications, as well as supplemental materials on developing, maintaining, and publicizing a vasectomy service.^76^ The second document provides guidance for organizing and conducting training in NSV. In many areas, NSV services are provided as part of a team effort; thus, this course included instructions for training vasectomy assistants as well as physicians.^72^

EngenderHealth published a checklist of the minimum number and types of medical instruments and supplies needed for provision of hormonal implants, IUDs, female sterilization, and vasectomy,^75^ which could be informative in future vasectomy programs and family planning/reproductive health costing studies.

The Johns Hopkins Information and Knowledge for Optimal Health (INFO) Project created a set of tools, checklists, and tables for program implementers and family planning providers to (1) counsel men about vasectomy, (2) identify men with conditions that require a delay or special consideration before vasectomy provision, and (3) explain to men what they should do before and after the vasectomy.^74^

Our search did not identify any tools or guidelines to provide couples' counseling, but one article previously discussed referenced use of a group counseling technique involving couples.^61^ Another INFO Project toolkit informs family planning/reproductive health program managers about the benefits of vasectomy and considerations for vasectomy integration.^76^

### Enabling Environment

Sociocultural, economic, and policy factors influence health services as well as social norms related to family planning in general and to va-sectomy in particular. An enabling environment for vasectomy requires engagement of governments, communities, and civil societies to support and advocate gender-equitable norms, accountability, evidence-based policies, and high-quality vasectomy services.

#### Barriers to Vasectomy Adoption

**Social norms against vasectomy.** In many studies, vasectomy was viewed by people as the least preferred contraceptive method and was often used only as a “last resort” for women who have experienced side effects from hormonal methods or who might have potential health risks with another pregnancy, or for a couple who has reached or exceeded their desired family size.^22^^,^^24^^,^^25^^,^^36^ Across studies, the most commonly mentioned misperceptions about vasectomy among both men and women were (1) a man would become physically weaker after having a vasectomy; (2) a man would be unable to function sexually after having a vasectomy (e.g., would be unable to have an erection or would be impotent, would have reduced sexual desire, would be incapable of enjoying sex or satisfying a woman, or would have impaired ejaculation); and (3) vasectomy was the same as castration.

Many people view vasectomy as a “last resort” for women who have experienced side effects with hormonal methods or other problems.

As mentioned earlier, the literature frequently cited prospective patient and provider reluctance to adopt vasectomy. This lack of acceptance among prospective clients and trusted health care providers perpetuates the intransigent social norm that family planning is a woman's duty.^15^^,^^16^^,^^18^^,^^31^

#### Facilitators for Vasectomy Services

Identifying appropriate areas in national and regional family planning strategies to highlight and support vasectomy integration is essential in formalizing support for the method. The literature we reviewed did not include effective messaging around economic benefits or direct links between programmatic activities and resulting policies. But the literature did include several program documents that described activities that were implemented with the goal of creating a more enabling environment for vasectomy adoption. Below, we combine different program activities around this goal.

**Multi-level engagement.** Gaining the support of governments and religious and community leaders and institutions can influence public attitudes toward public health campaigns, including vasectomy uptake. As an example, Simbar attributes Iran's increased religious and political support of family planning programming over the last decade as a fundamental component to increased contraceptive uptake in the country.^77^ Currently, Iran's vasectomy program is moderately robust with about 30,000 vasectomies provided annually^78^ and may provide a model for other countries in the region. Unfortunately, media reports from 2014 suggest that there was legislation passed by the government to ban vasectomy as a means to increase population.^79^ We are unaware of the current availability of the method in the country.

In Tanzania, the ACQUIRE Project identified Seventh-Day Adventists as advocates of all forms of contraception, including vasectomy, who even included information on contraception in their sermons.^29^ The Heri Seventh Day Adventist Mission Hospital in Tanzania, a focal point of the project's vasectomy promotion and training activities, provided vasectomy services and educational seminars about the benefits of contraception.^29^^,^^30^ This hospital became a regional center of excellence in NSV and provided the majority of vasectomies over a 6-year period in the Kigoma region.

In Bangladesh, the ACQUIRE Project produced a book entitled *Family Planning in the Eyes of Islam,* designed to engage influential *imams* (Muslim religious leaders) in family planning, with a focus on LAPMs. This book situated the role of family planning in Islam and the stance taken on family planning in the Qur'an and Hadith, Islam's 2 foremost sacred texts. In addition, the ACQUIRE Project sponsored interactive community forums, largely held in rural areas of Bangladesh, that brought together *imams*, teachers, businessmen, local politicians, and local family planning service providers to discuss family planning and the important role of LAPMs.^78^

**Gender transformative messaging.** The “Get a Permanent Smile” campaign in Bangladesh and Ghana (as previously described) addressed the myths associated with vasectomy, particularly related to men's interest in and knowledge of family planning.^78^ The program created posters and television commercials that contained the message “My husband is best,” which was highly regarded in the community. Men liked the fact that the materials clearly illustrated their role in family planning decision making and the notion that a wife would value the husband's involvement. The materials challenged frequently cited concerns about vasectomy, promoted vasectomy in the communities, and highlighted couples' shared decision making.^81^

In Honduras, the “Permanent Smiles” campaign aimed to reposition vasectomy as a simple and effective male method of family planning.^78^ Key messages emphasized that vasectomy would have no negative effects on couples' relationships and that vasectomy does not affect a man's sexual performance.

In Ghana, the ACQUIRE Project's vasectomy promotion included an emphasis on the benefits of vasectomy and promoted “satisfied users” through testimonials. Vasectomy was promoted as a contraceptive method that gives a man the ability to care for his partner and children while offering the freedom to enjoy life.^54^

## DISCUSSION AND RECOMMENDATIONS

This review identified factors that facilitate va-sectomy integration into national family planning agendas from the experiences and evidence of recent vasectomy programs in low-resource settings. Vasectomy, like other contraceptive methods, benefits from well-integrated demand generation activities and adequately trained providers. Supportive policies are directly linked to the potential for va-sectomy uptake. Government health agencies (if they have not done so already) must establish policies and political infrastructure that strategically engage and include men in a comprehensive reproductive health agenda, without undermining the gains made in improving access to family planning for women. Policies that empower women and men to be supportive partners, continual family planning users, and reproductive health advocates lay a solid foundation for future vasectomy programs.

Vasectomy, like other contraceptive methods, benefits from well-integrated demand generation activities and adequately trained providers.

Unfortunately, current approaches to vasectomy integration are focused on the “quick win.” These approaches advocate the benefits of vasectomy to men and couples ready for a vasectomy right now. We suggest that vasectomy can be used to address the fundamental gap between reproductive health programming and men, a gap that exists in both high- and low-resource settings.^81^ These approaches would include educating men, including young men, on the range of methods available in their communities, their potential side effects, and their effectiveness. Research has shown that engaging men in family planning and reproductive health increases couple communication, facilitates male involvement in child care, and improves relationships.^82^^–^^85^ Maintaining the status quo of male exclusion from reproductive health services stagnates development and reinforces negative gender norms (i.e., use of contraception is a woman's responsibility). Therefore, increasing men's reproductive health lexicon and addressing existing gender normative barriers can help both families to achieve their reproductive goals and countries to achieve their national family planning goals.

Engaging men in family planning increases couple communication, facilitates male involvement in child care, and improves relationships.

Limited human resources continually limit the service provision, quality of care, and accuracy of clinical data. When considering vasectomy integration, governments should investigate the appropriateness of using health facility staff that are not physicians. It has been suggested that countries where nurses are already performing adult male circumcisions would be skilled enough to take on vasectomy provision.^86^ This is not to suggest that vasectomy clients would necessarily be the same men at the same time as circumcision clients, because circumcision clients^87^ may not be in the same life stage as vasectomy clients.^41^ Also, this would require clear delineation in counseling and promotion of the 2 methods.

Three reasons make a case for exploring this task-shifting option:

1. The 2 procedures are similar in surgical complexity.
2. Nurses who are already performing adult circumcisions have demonstrated the necessary surgical talent.
3. These nurses are accustomed to dealing with men in a reproductive health context.

The benefits of strong intake counseling cannot be understated for vasectomy services. Vasectomy's global history^88^ and the mandate of informed choice should be considered when training health facility staff to counsel clients. Clear articulation of vasectomy as a permanent method should be included in counseling. Rates of dissatisfaction and/or regret with vasectomy range between 1% and 2%,^89^ and between 3% and 6% of men request reversals.^90^ In most low-resource countries, opportunities for a vasectomy reversal is not likely an option. Vasectomy counseling should also address emotional issues associated with the loss of fertility and the end of a couple's reproductive life. The “maturational loss” associated with this change is well documented but seldom investigated in vasectomy research.^91^

Universally, the studies and programmatic reports included in this review reflect positive and proactive approaches to vasectomy service provision. Unfortunately, there are also examples of misuse and abusive implementation in the past.^88^ Reflecting upon this history is key for program implementers, funders, and policy makers. It should also reinforce the principle of informed choice of family planning methods. Informed choice is one aspect of the growing “rights-based approach” that is currently being integrated into program planning that is being championed by global funders and initiatives including FP2020.^92^

The program literature provides examples of channels and activities that changed perceptions of vasectomy and integration of men into reproductive health services. Countering misperceptions across multiple media channels was found to be effective at increasing vasectomy demand. Examples and materials to support vasectomy integration are well established. Use of testimonials, media campaigns, and strategic timing of service delivery was found to facilitate uptake, while training tools have been well established and easily available. At the grassroots level, cadres of existing vasectomy providers are linking themselves with physicians in low-resource settings.^93^ Training these providers in low-resource settings and providing them with the necessary equipment is important, but their small scale has limited impact on national contraceptive prevalence rates or on the associated benefits of integrating vasectomy into a national family planning agenda.

Countering misperceptions across multiple media channels has been effective at increasing vasectomy demand.

In conjunction with these somewhat grassroots efforts, World Vasectomy Day—the global campaign that fosters discussions about men's role in reproductive health—held its fourth annual event on Friday, November 18, 2016, in Kenya.^93^ Momentum for vasectomy integration is rising and now the challenge is to appropriately pivot the focus of family planning and reproductive health services to include men in meaningful and impactful ways.

## Supplementary Material

supplementary material

## Notes

**Peer Reviewed**
