# Supplementary material for: A Review of 10 Years of Vasectomy Programming and Research in Low-Resource Settings
Source: Glob Health Sci Pract. 2016 Dec 23;4(4):647–60. doi: 10.9745/GHSP-D-16-00235 (PMC5199180; doi:10.9745/GHSP-D-16-00235)
Supplement: supplementary material [file GHSP-D-16-00235_index.html]

Supplement to A Review of 10 Years of Vasectomy Programming and Research in Low-Resource Settings | Global Health: Science and Practice

## Supplemental material

- Text so1, PDF - Text so1, PDF
