# Supplementary material for: A Review of 10 Years of Vasectomy Programming and Research in Low-Resource Settings
Source: Glob Health Sci Pract. 2016 Dec 23;4(4):647–60. doi: 10.9745/GHSP-D-16-00235 (PMC5199180; doi:10.9745/GHSP-D-16-00235)
Supplement: supplementary material [file 16-00235-Shattuck-Supplementary_material.pdf]

Shattuck D, Perry B, Packer C, Chin Quee D. A review of 10 years of vasectomy programming and research in low-resource settings. Glob Health Sci Pract. 2016;4(4). http://dx.doi.org/10.9745/GHSP-D-16-00235

Supplementary Material. Documents Included in the Vasectomy Review, by Region

Source	Title	Project	Country	Demand	Supply	Enabling Environment	Summary
**Global**							
EngenderHealth 2007	No-scalpel vasectomy curriculum: A training course for vasectomy providers and assistants, 2nd edition. Participant handbook	ACQUIRE Project	Global		X		This curriculum is a clinical skills training course designed to train physicians and vasectomy assistants to provide safe, effective NSV services. Besides containing instructions on providing the NSV procedure, this course also contains information on counseling, informed consent, infection prevention, and management of complications, as well as supplemental materials on developing, maintaining, and publicizing a vasectomy service.
EngenderHealth 2007	No-scalpel vasectomy curriculum: A training course for vasectomy providers and assistants, 2nd edition. Trainer's manual	ACQUIRE Project	Global		X		This curriculum on NSV is a clinical course designed to train physicians and vasectomy assistants to provide NSV. This course emphasizes the information needed to provide safe and effective NSV services and may require extensive practice time. It assumes that participants will bring skills, knowledge, and self-motivation to the training. In many areas, NSV services are provided as part of a team effort; thus, this course includes instructions for training vasectomy assistants.
EngenderHealth 2013	Instruments and supplies needed to provide clinical methods of family planning	RESPOND Project	Global		X		This is a checklist of the minimum number and types of medical instruments and supplies that EngenderHealth recommends as needed for provision of each of the four clinical methods of family planning (hormonal implants, IUDs, female sterilization, and vasectomy).
Family Health International 2008	Improving provision of vasectomy	Contraceptive and Reproductive Health Technologies Research and Utilization Program	Global	X			Provides specific recommendations for improving demand creation and user satisfaction.
Glasier 2010	Acceptability of contraception for men: a review	-	Global	X			Review that describes acceptability of male contraception in general, including vasectomy, but is more focused on hormonal male contraception.

Jacobstein 2007	Vasectomy: the unfinished agenda	ACQUIRE Project	Global	X	X	X	An overview of supply, demand, and policy barriers to vasectomy uptake as well as a summary of recommendations generated based on previous vasectomy work.
John Snow Inc. 2010	Using quantification to support introduction and expansion of long-acting and permanent methods of contraception	RESPOND Project	Global		X	X	Guidelines and recommendations for "quantification" — forecasting and supply planning — to address the challenge of providing access to provider-dependent FP services.
Kols 2008	Vasectomy: Reaching out to new users	Information and Knowledge for Optimal Health Project	Global		X		A toolkit to inform FP/RH program managers about the benefits of vasectomy and considerations for vasectomy integration.
Lande 2008	Vasectomy: Tools for providers	Information and Knowledge for Optimal Health Project	Global		X		A toolkit for FP/RH counselors to inform vasectomy clients about the procedure and dispel potential myths/rumors.
Pile & Barone, 2009	Demographics of Vasectomy - USA and International	-	United States and Global	X			Review that describes trends and prevalence of vasectomy in all regions of the world and includes demographics of users.
**Sub-Saharan Africa**							
Adongo 2014	"If you do vasectomy and come back here weak, I will divorce you": a qualitative study of community perceptions about vasectomy in Southern Ghana		Ghana	X			Qualitative study with male and female community members, community health officers, community health volunteers, and district and regional health managers, which explores the social and cultural factors that affect vasectomy uptake in southern Ghana.
Akafuah 2008	Attitudes toward and use of knowledge about family planning among Ghanaian men		Ghana	X			Exploratory study to examine sociocultural factors related to knowledge, attitudes about, and practice of FP among a convenience sample of 200 men in Ghana.
Akpamu 2010	Knowledge and acceptance of 'vasectomy as a method of contraception' amongst literate married men in Ekpoma, Nigeria		Nigeria	X			Study that investigates the knowledge and acceptance of vasectomy as a male contraceptive method in Ekpoma, Edo State, Nigeria, among 350 literate, married men.
Alemayehu 2012	Factors associated with utilization of long acting and permanent contraceptive methods among married women of reproductive age in Mekelle town, Tigray region, north Ethiopia		Ethiopia	X			A cross-sectional community-based survey and qualitative component conducted with married men and women to assess factors associated with utilization of LAPMs.
Babalola 2013	Views on family planning and long-acting and permanent methods: insights from Malawi	RESPOND Project	Malawi	X			A project brief describing the RESPOND Project's results from and recommendations based on qualitative research conducted in Malawi among married and unmarried men and women, FP providers, and key

							informants to gain insights into the factors that may constrain the use of LAPMs.
Babalola 2012	Factors underlying the use of long-acting and permanent family planning methods in Nigeria: a qualitative study	RESPOND Project	Nigeria	X			A full report describing the RESPOND Project's results from and recommendations based on qualitative research conducted in Nigeria among married and unmarried men and women, FP providers, and key informants to gain insights into the factors that may constrain the use of LAPMs.
Babalola 2013	Views on family planning and long-acting and permanent methods: insights from Nigeria	RESPOND Project	Nigeria	X			A project brief describing the RESPOND Project's results from and recommendations based on qualitative research conducted in Nigeria among married and unmarried men and women, FP providers, and key informants to gain insights into the factors that may constrain the use of LAPMs.
Bunce 2007	Factors affecting vasectomy acceptability in Tanzania	ACQUIRE Project	Tanzania	X		X	Qualitative study among potential and actual vasectomy users and their wives describing factors affecting vasectomy acceptability in a region where ACQUIRE was working to focus on male RH services.
Cisek 2008	Revitalizing underutilized family planning methods: assessing the impact of an integrated supply-demand vasectomy initiative in Ghana	ACQUIRE Project	Ghana	X	X	X	Summary report of ACQUIRE's implementation of the SDA model to increase supply and demand of vasectomy in Ghana.
Davis 2009	Final feasibility evaluation for no-scalpel vasectomy in Rwanda	Capacity Project	Rwanda		X		Reports the activities and outcomes from training physicians and nurses in Rwanda on the use of NSV. The document also provides a service map/clinic flow diagram that could assist future clinics in their vasectomy integration plans.
Davis 2010	Successful no-scalpel vasectomy pilot program in Rwanda	Capacity Project	Rwanda	X	X		Technical brief provides details about user characteristics along with their motivations for getting a vasectomy and pilot training program outcomes.
de Vries 2009	Repositioning family planning: Rwanda's no-scalpel vasectomy program	Capacity project	Rwanda	X	X		Summary of the activities conducted as part of the Capacity Project to introduce vasectomy services in select Rwandan clinics. Includes motivations for getting vasectomy and satisfaction with services.
Ebeigbe 2011	Vasectomy: a survey of attitudes, counseling patterns and acceptance among Nigerian resident gynaecologists	-	Nigeria	X	X		A cross-sectional survey conducted with 104 resident OBGYN doctors in Nigeria conducted to determine the level of knowledge of, attitudes toward, counseling patterns regarding, and acceptance of vasectomy.
FHI 360 2011	Rwanda takes no-scalpel vasectomy nationwide	PROGRESS	Rwanda		X		Description of activities conducted to scale up vasectomy services — particularly the use of NSV with FI and thermal cautery — in Rwanda.
FHI 360 2013	No-scalpel vasectomy: scale-up. Approach in Rwanda shows promise	PROGRESS	Rwanda	X	X		A description of the monitoring efforts conducted by the Rwanda MOH and FHI 360 to understand institutional, structural, and individual factors influencing the choice of vasectomy in Rwanda and to improve quality and efficiency of the nationwide program.

Frajzyngier 2006	Factors affecting vasectomy acceptability in the Kigoma region of Tanzania	ACQUIRE Project	Tanzania	X		X	In-depth study with partners of and men who had and did not have vasectomies, key opinion leaders, and service statistics to explore the variables that affect the decision-making process and to assess leaders' influence on vasectomy use and men's RH.
Kabenyi 2014	Barriers to male involvement in contraceptive uptake and reproductive health services: a qualitative study of men and women's perceptions in two rural districts in Uganda	-	Uganda	X			A qualitative study with men, women, and key informants (government and community leaders) to examine obstacles to men's support and uptake of modern contraceptives in Bugiri and Mpigi districts, Uganda.
Labrecque 2013	Strengthening vasectomy services in Rwanda: introduction of thermal cautery with fascial interposition	PROGRESS	Rwanda		X		Description of a provider training program on NSV with cautery combined with FI, along with associated costs of materials and length of training activities.
Nduka 2014	Perception of antenatal clinic attendees towards voluntary surgical contraception in a Nigerian Tertiary hospital	-	Nigeria	X			A study to explore knowledge and attitudes toward voluntary surgical contraception among 540 married and unmarried female antenatal clinic patients in Nigeria.
Ochieng, 2014	Determinants of readiness to undergo vasectomy, a family planning method for men in Busia County, Kenya	-	Kenya	X			A master's student's research project that analyzes socioeconomic and service-related factors associated with men's willingness to use vasectomy. Includes men who had and have not had vasectomies.
Odu 2006	Men's knowledge of and attitude with respect to family planning in a sub-urban Nigerian community	-	Nigeria	X			A descriptive, cross-sectional study to determine knowledge of and attitudes toward FP among 360 married and unmarried men in Ilorin, Nigeria.
Okunlola 2009	Awareness and practice of vasectomy among married male health workers at the University College Hospital, Ibadan, Nigeria	-	Nigeria	X			A descriptive, cross-sectional study to determine the awareness and practice of vasectomy among 250 male health workers at the University College Hospital, Ibadan.
Onasoga 2013	Knowledge and attitude of men towards vasectomy as a family planning method in Edo State, Nigeria	-	Nigeria	X			A descriptive study to assess the level of knowledge of vasectomy and determine the attitudes and factors influencing attitudes toward vasectomy among 136 men in Edo State, Nigeria.
Owusu-Asubonteng 2012	Trend, client profile and surgical features of vasectomy in Ghana	-	Ghana	X			Retrospective review of 271 vasectomies performed between January 2000 and December 2009 in three health care facilities to examine socio-demographic and reproductive characteristics of vasectomy users.
Rajani 2006	'Get a Permanent Smile' — increasing awareness of, access to, and utilization of vasectomy services in Ghana	ACQUIRE Project	Ghana	X	X	X	Description of the various activities conducted in the "Permanent Smiles" campaign in Ghana, as well as specific activities related to enhancing supply-side issues and demand-side concerns.

Shattuck 2014	Who choses vasectomy in Rwanda? Survey data from couples who chose vasectomy, 2010-2012	-	Rwanda	X			This cross-sectional descriptive study describes vasectomy clients (n=316) and their wives (n=300) from 15 randomly selected hospitals in Rwanda.
Trollip 2009	Vasectomy under local anaesthesia performed free of charge as a family planning service: complications and results	-	South Africa		X		This study evaluates the safety and efficacy of vasectomy performed under local anesthesia by junior physicians at a secondary level hospital as part of a free FP service.
**Asia and the Middle East**							
Azmat 2012	Barriers and perceptions regarding different contraceptives and family planning practices amongst men and women of reproductive age in rural Pakistan: a qualitative study		Pakistan	X			A qualitative study among men and women in rural Pakistan to understand the barriers to FP, knowledge of FP, perceptions regarding FP, quality of care, and free FP services.
Bathula 2013	Social stigma associated with vasectomy among females of Thullur Mandal in Guntur District		India	X			A cross-sectional study with 150 female postnatal patients in Andhra Pradesh, India, to explore the reasons why women opt for female sterilization rather than vasectomy.
Cui 2010	Factors influencing the declining trend of vasectomy in Sichuan, China	-	China	X			A qualitative study among FP providers and wives of and men who had and did not have vasectomies, to describe the reasons for the declining trend of vasectomy in a region of China.
Dilbaz 2007	Outcome of vasectomies performed at a Turkish metropolitan maternity hospital	-	Turkey	X	X		A study to determine prospectively the outcome of vasectomies performed by two trained surgeons over nine months. Includes user characteristics, complication rates, and follow-up rates.
Garg 2013	Nonscalpel vasectomy as family planning method: a battle yet to be conquered	-	India	X			A cross-sectional study among 428 married men with at least one child in North India to understand the barriers to using NSV related to knowledge, attitudes, and sources of information.
Garima 2013	Trends of utilization of family planning methods at district hospital of Madhya Pradesh: a retrospective study	-	India	X			This retrospective study analyzes the trends of utilization of different FP methods, including vasectomy, in relation to socio-demographic factors at a district hospital in the last five years.
Gunenc 2009	Opinions concerning male and female sterilisation in Turkey	-	Turkey	X			A descriptive cross-sectional study to determine the attitudes of women and men regarding male and female sterilization among 1,211 women of reproductive age and their husbands (n=1174).
Hall 2008	Social and logistical barriers to the use of reversible contraception among women in a rural Indian village	-	India	X			A qualitative study to understand women's preference for female sterilization and attitudes toward vasectomy and reversible contraception among women in western India.
Jabeen 2006	Psychosocial factors and male sterilization	-	Pakistan	X			A study looking at the demographic profile of vasectomy clients and the psychosocial factors motivating them to use vasectomy.
Keramat 2011	Barriers and facilitators affecting vasectomy acceptability (a multi	-	Iran	X			A study that describes factors associated with acceptability of vasectomy among partners of and men who had and did not have vasectomies.

Shattuck D, Perry B, Packer C, Chin Quee D. A review of 10 years of vasectomy programming and research in low-resource settings. Glob Health Sci Pract. 2016;4(4). http://dx.doi.org/10.9745/GHSP-D-16-00235

	stages study in a sample from north eastern of Iran), 2005-2007						
Kumar 2007	Men's perspective on non-scalpel vasectomy in rural Kerala	-	India	X			A cross-sectional study that assesses the knowledge, attitudes, and behavior related to NSV among 661 married men with at least one child in Kerala, India.
Labrecque 2005	Vasectomy surgical techniques in South and South East Asia	ACQUIRE Project	Cambodia, Thailand, India, Nepal, and Bangladesh		X	X	This study summarizes the surgical vasectomy techniques currently used in five Asian countries and evaluates the facilitating and limiting factors to introduction and assessment of FI and thermal cautery in these countries.
Mahapatra 2014	Assessment of knowledge and perception regarding male sterilization (non-scalpel vasectomy) among community health workers in Jharkhand, India	-	India	X	X		A cross-sectional study that assesses the knowledge and perception of 200 CHWs regarding vasectomy in the Simdega district of Jharkhand, India.
Mehra 2013	Orientations: a capacity building tool for improving knowledge and perception of health workers regarding non scalpel vasectomy	-	India	X	X		This study assesses the knowledge of CHWs regarding NSV and the effect of orientation in improving their knowledge.
Nagarajappa 2005	A study on knowledge of married men on no-scalpel vasectomy	-	India	X			A cross-section study to assess knowledge of NSV and compare knowledge with demographic variables among 200 married men with at least one child in Bangalore, India.
Nishtar 2013	Myths and fallacies about male contraceptive methods: a qualitative study amongst married youth in slums of Karachi, Pakistan	-	Pakistan	X			A qualitative study to explore perceptions regarding myths and fallacies related to male contraception among married young men and women (18-24 years) in Pakistan.
Padmadas 2014	Do mobile family planning clinics facilitate vasectomy use in Nepal?	-	Nepal		X		Assessment of the impact of mobile clinics in improving access and uptake of vasectomy services in remote areas.
Sahin 2008	Male university students' views, attitudes and behaviors towards family planning and emergency contraception in Turkey	-	Turkey	X			A descriptive study with 278 male university students to determine their views, attitudes and behavior towards FP and emergency contraception in Turkey.
Sahin 2008	Male participation in contraception in an eastern province of Turkey	-	Turkey	X			A descriptive study to determine 801 men's knowledge, attitudes, and practice toward FP in a northeastern province of Turkey.
Scott 2011	Factors affecting acceptance of vasectomy in Uttar Pradesh: insights from community-based, participatory qualitative research	-	India	X			A participatory ethnographic evaluation research study with men and women to understand the reasons for the low prevalence of vasectomy in Uttar Pradesh, India, and to contribute to developing an approach for increasing demand.
Simbar 2012	Achievements of the Iranian family planning programmes 1956-2006	-	Iran	X	X	X	A review of the FP programs in Iran and their achievements during the latter half of the 20th century. This paper proposes potential successful strategies for health promotion and behavior change.

Shattuck D, Perry B, Packer C, Chin Quee D. A review of 10 years of vasectomy programming and research in low-resource settings. Glob Health Sci Pract. 2016;4(4). http://dx.doi.org/10.9745/GHSP-D-16-00235

Singh 2014	Increasing male participation in the uptake of vasectomy services	RESPOND Project	India	X	X	X	Summary of interventions conducted as part of the RESPOND Project in India to address misperceptions about the vasectomy procedure among men and women and to ensure quality of services provided.
Stover 2007	Long-term and permanent methods of family planning in Bangladesh	Global Health Technical Assistance Project (GH Tech)/ACQUIRE	Bangladesh	X	X	X	Reviews the status of and potential for strengthening support for LAPMs throughout the public, nongovernmental, and private sectors of Bangladesh.
Taylor 2008	Revitalizing underutilized family planning methods: using communications and community engagement to stimulate demand for vasectomy in Bangladesh	ACQUIRE Project	Bangladesh	X	X	X	An overview of the SDA Program Model for FP/RH Service Delivery to coordinate and synchronize these mutually reinforcing components affecting the acceptance of FP services.
The RESPOND Project 2014	End-of-project evaluation of the RESPOND No-Scalpel Vasectomy Initiative in Uttar Pradesh and Jharkhand States, India	RESPOND Project	India	X	X	X	Reports the results from technical assistance offered through the RESPOND Project in Uttar Pradesh and Jharkhand, India, according to the SEED Programming Model.
Tuladhar 2008	Awareness and practice of family planning methods in women attending Gyne OPD at Nepal Medical College Teaching Hospital	-	Nepal	X			A cross-sectional descriptive study of awareness and practice of FP methods among 200 female gynecology patients in Nepal.
Valsangkar 2012	Predictors of no-scalpel vasectomy acceptance in Karimnagar District, Andhra Pradesh	-	India	X			A community-based, case-control study (men who had and did not have vasectomies) conducted to elucidate the reasons for higher acceptance of NSV in Karimnagar District compared to state and national rates.
Yahner 2012	Using an employer-based approach to increase support for and provision of long-acting and permanent methods of contraception: the India experience	RESPOND Project	India	X			Reports the activities and outcomes of an employer-based FP/RH promotion intervention in India.
Yinger 2013	Views on family planning and long-acting and permanent methods: insights from Cambodia	RESPOND Project	Cambodia	X			A project brief describing the RESPOND Project's results from and recommendations based on qualitative research conducted in Cambodia among married and unmarried men and women, FP providers, and key informants to gain insights into the factors that may constrain the use of LAPMs.
**Latin America and the Caribbean**							
de Rodriguez 2005	Expanding access to vasectomy services in the Ministry of Health of Guatemala	FRONTIERS Project	Guatemala		X		Reports the activities conducted as part of the FRONTIERS Project in Guatemala to introduce NSV services in selected clinics.

Manhoso 2005	Men's experiences of vasectomy in the Brazilian Public Health Service	-	Brazil	X			Qualitative study that describes the experiences of a group of 20 men who had had vasectomies, including motivations to get a vasectomy and satisfaction with services.
Marchi 2008	Contraceptive methods with male participation: a perspective of Brazilian couples	-	Brazil	X			A qualitative study with 20 couples who had requested vasectomy to assess perspectives on male participation in FP and contraceptive methods available to men in Campina, Brazil.
Marchi 2010	Vasectomy within the public health services in Campinas, Sao Paulo, Brazil	-	Brazil	X			Descriptive study that describes characteristics of 202 men who had a vasectomy in the public health network between 1998 and 2004.
Taylor 2008	Revitalizing underutilized family planning methods: using communications and community engagement to stimulate demand for vasectomy in Honduras	ACQUIRE Project	Honduras	X	X	X	Summary of demand-side interventions initiated in late 2004 aimed to reposition vasectomy as a simple and effective male method of FP in Honduras.
The FRONTIERS Project 2007	On-site training and outreach for introducing vasectomy services	FRONTIERS Project	Guatemala		X		Summarizes the results of the FRONTIERS Project in Guatemala to introduce NSV services in selected clinics.
Vernon 2007	Introducing sustainable vasectomy services in Guatemala	FRONTIERS Project	Guatemala		X		This document describes the efforts of the FRONTIERS Project in Guatemala to develop, test, and evaluate a model for the introduction of sustainable NSV services in MOH hospitals.
**Multiple countries from different regions**							
Wickstrom 2013	Approaches to mobile outreach services for family planning: a descriptive inquiry in Malawi, Nepal, and Tanzania	RESPOND Project	Malawi, Nepal, Tanzania		X		Documents the role of mobile outreach to fulfill FP/RH client needs in Malawi, Nepal, and Tanzania.
Seamans 2007	Modelling cost-effectiveness of different vasectomy methods in India, Kenya, and Mexico	HealthTech IV	India, Kenya and Mexico		X		Compares the cost-effectiveness of different vas occlusion methods.
